# Therapeutic challenges in relapsing cutaneous and visceral leishmaniasis caused by *Leishmania* (*Mundinia*) *martiniquensis* in patients with advanced HIV disease from Southern Thailand

**DOI:** 10.1186/s41182-026-00931-9

**Published:** 2026-02-23

**Authors:** Kobpat Phadungsaksawasdi, Nopporn Songumpai, Chanutta Swasdivanich, Pasinee Rongngern, Tanaporn Borriboon, Chatuthanai Savigamin, Narisa Brownell, Kanyarat Kraivichian, Nopadon Noppakun, Padet Siriyasatien, Pravit Asawanonda, Kanok Preativatanyou

**Affiliations:** 1 Division of Dermatology, Department of Medicine, Faculty of Medicine, Chulalongkorn University, Bangkok, Thailand; 2 Center of Excellence in Vector Biology and Vector-Borne Disease, Faculty of Medicine, Chulalongkorn University, Bangkok, Thailand; 3 Department of Parasitology, Faculty of Medicine, Chulalongkorn University, Bangkok, Thailand; 4 Division of Infectious Diseases, Department of Internal Medicine, Hatyai Hospital, Songkhla, Thailand; 5 Division of Dermatology, Department of Internal Medicine, Hatyai Hospital, Songkhla, Thailand; 6 Dermatopathology Unit, Institute of Dermatology, Bangkok, Thailand; 7 Division of Rheumatology, Department of Medicine, Johns Hopkins University, Baltimore, MD USA

**Keywords:** *Leishmania martiniquensis*, *Mundinia*, Non-ulcerated lesions, Visceral leishmaniasis, Diffuse cutaneous leishmaniasis, Relapse

## Abstract

**Background:**

Autochthonous leishmaniasis has become increasingly recognized in Thailand, with *Leishmania* (*Mundinia*) *martiniquensis* identified as the predominant species, particularly among immunocompromised individuals. Infected immunosuppressed patients often present with complex clinical features, which can delay diagnosis and complicate treatment. Given the limited clinical data available and emerging reports of resistant infections, improved awareness, prompt diagnosis, and optimized management strategies are urgently needed to address this underrecognized pathogen in Thailand.

**Case presentation:**

We report two patients with advanced HIV disease (AHD) from Songkhla Province, Southern Thailand, who developed chronic diffuse cutaneous leishmaniasis characterized by widespread non-ulcerative papulonodular lesions that progressed to visceral involvement. Histopathological examination of the skin nodules showed prominent dermal fibrosis with infiltration by macrophages heavily parasitized with kinetoplast-containing amastigotes, consistent with cutaneous leishmaniasis. Molecular analyses identified *L. martiniquensis* as the causative agent in both cases. The parasite strains (WHO codes: MHOM/TH/2022/CULE7.1 and MHOM/TH/2022/CULE7.2) were successfully isolated from the bone marrow and cutaneous biopsy of the second patient before treatment. Furthermore, the parasite was isolated again from a cutaneous biopsy of the same patient after relapse, designated MHOM/TH/2023/CULE8. Due to the high costs of liposomal amphotericin B and the unavailability of miltefosine in Thailand, contrary to the WHO guideline recommending these as first-line therapy, patients received intravenous amphotericin B deoxycholate (AmB-D) combined with oral itraconazole. Despite repeated treatment with AmB-D and itraconazole, both patients relapsed, and Case 1 died. This raises concerns about drug resistance.

**Conclusions:**

These cases illustrate complex cutaneous manifestations and therapeutic challenges of relapsing diffuse cutaneous and visceral leishmaniasis caused by *L. martiniquensis* in patients with AHD from Southern Thailand. The persistence and relapse despite AmB-D therapy raise concerns about emerging drug-resistant strains and underscore the need for enhanced surveillance, parasite isolation, and optimized treatment strategies for this neglected pathogen. Moreover, this report expands the understanding of the cutaneous spectrum of *L. martiniquensis* in patients with AHD, emphasizing the importance of including leishmaniasis in the differential diagnosis of complex skin diseases among immunosuppressed individuals, particularly in endemic areas.

## Background

Leishmaniasis is a neglected vector-borne disease caused by obligate intracellular parasites of the genus *Leishmania*, which are known to be transmitted by infected female sand flies. The disease manifests in three primary clinical forms, cutaneous leishmaniasis (CL), mucocutaneous leishmaniasis (MCL), and visceral leishmaniasis (VL), depending on the infecting species and the host's immune status [1]. Over 50 species of *Leishmania* have been identified, with at least 20 known to cause human disease [2]. Taxonomically, these parasites are classified into four subgenera: *Leishmania*, *Viannia*, *Sauroleishmania*, and the recently described *Mundinia* [3]. Of note, *Leishmania martiniquensis* and* Leishmania orientalis*, members of the *Mundinia* subgenus, are the main causative agents of autochthonous leishmaniasis reported in Thailand and Myanmar [4–7].

In Thailand, autochthonous leishmaniasis has been reported predominantly from the northern and southern regions, with VL occurring mainly among immunosuppressed individuals, especially those with advanced HIV disease (AHD) [6]. These patients may present with severe concomitant manifestations, including diffuse CL, and often exhibit a limited therapeutic response to amphotericin B deoxycholate (AmB-D), a commonly used antileishmanial agent for treating VL in Thailand [6]. Here, we present two cases with AHD from Songkhla Province, Southern Thailand, who developed chronic diffuse CL progressing to concurrent VL caused by *L. martiniquensis*. Comprehensive clinical, histopathological, parasitological, and molecular data from these cases provide valuable insights into the complexity of leishmaniasis, highlighting the diagnostic and therapeutic challenges encountered in immunocompromised individuals.

## Case presentation

### Case 1

**Fig. 1:**
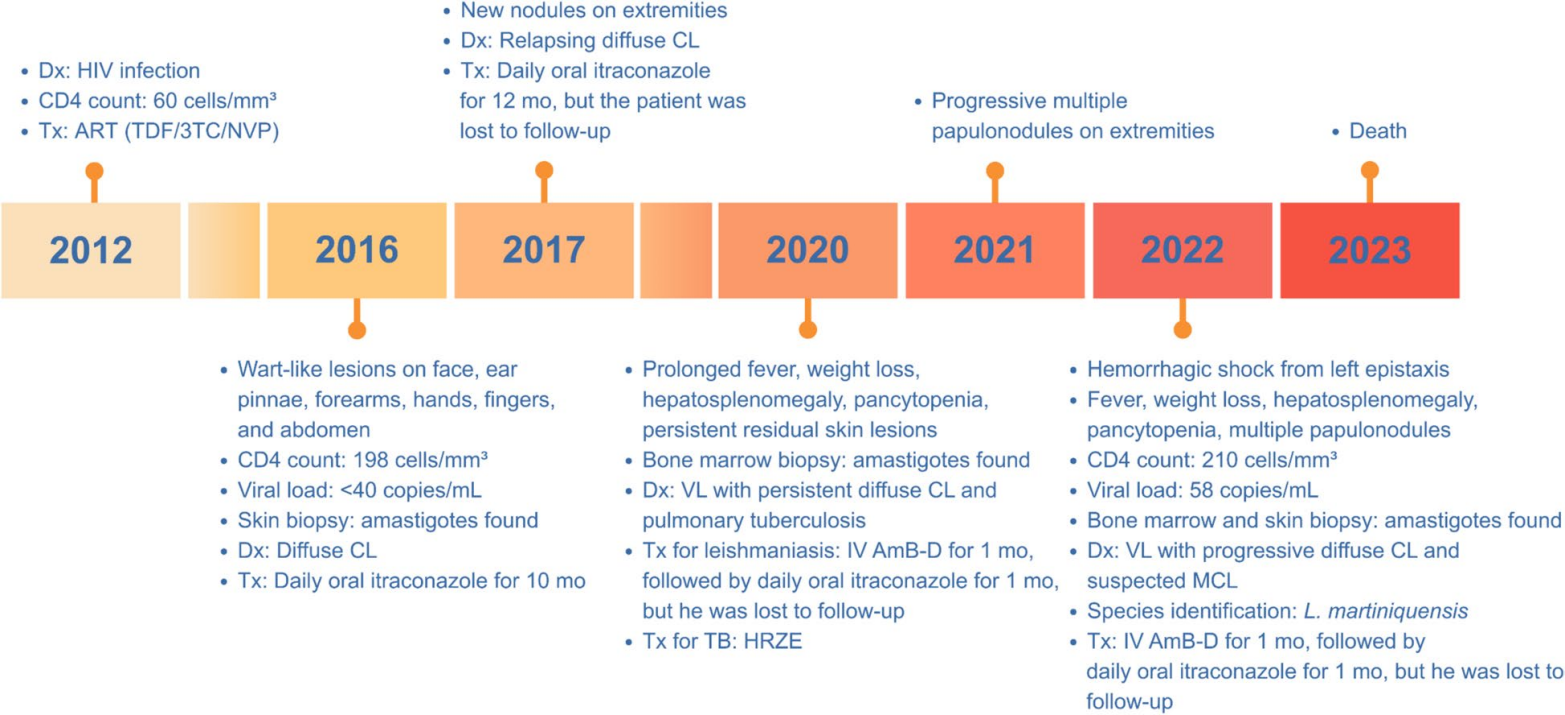
Key clinical timeline and management of Case 1 from 2012 to 2023. *Dx* Diagnosis, *Tx* Treatment, *IV* intravenous

In September 2022, a 55-year-old male with known underlying AHD (diagnosed in 2012 with an initial CD4 count: 60 cells/mm^3^), residing and working in a rubber tree plantation in Padang Besar Subdistrict, Sadao District, Songkhla Province, Southern Thailand, presented with multiple cutaneous papulonodular lesions and hemorrhagic shock, following an episode of left-sided epistaxis. He remained adherent to antiretroviral therapy (ART) consisting of tenofovir/lamivudine/nevirapine (TDF/3TC/NVP) since treatment initiation in 2012, with good compliance and no history of HIV drug resistance. His CD4 count in July 2016 was 198 cells/mm^3^ with a viral load < 40 copies/mL, and the most recent count in May 2022 was 210 cells/mm^3^ with a viral load of 58 copies/mL. Key clinical events and management of this case are summarized in Fig. 1.

**Fig. 2:**
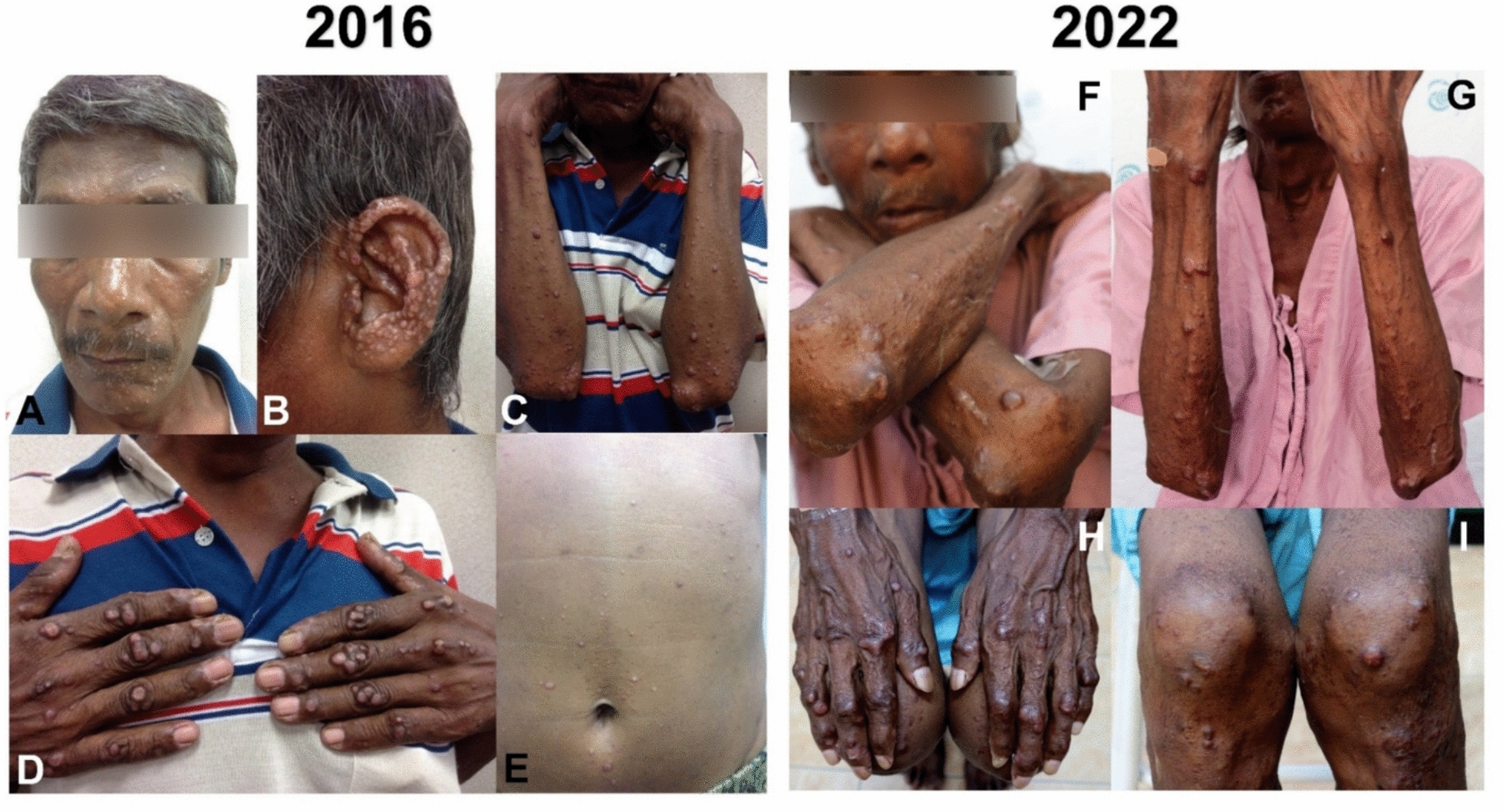
Cutaneous manifestations of Case 1 at initial (2016) and subsequent (2022) presentation. **A**–**E** 2016: Multiple discrete papules and nodules on face, auricle, extensor forearms, dorsal hands/fingers, and abdomen. **F**–**I** 2022: Multiple discrete papules and nodules on forearms, dorsal hands/fingers, and legs

His dermatological history dated back to 2016, when wart-like skin lesions first appeared on face, ear pinnae, forearms, hands, fingers, and abdomen (Fig. 2A–E). Cutaneous leishmaniasis was confirmed by microscopy from a skin biopsy. Due to underlying AHD-related immunosuppression, extended itraconazole therapy (200 mg daily) was selected. The patient showed significant improvement without adverse events after the initial 5 months, prompting dose reduction (100 mg daily) for an additional 5 months (total of 10 months). However, he did not return for follow-up medication. Three months later, new nodules appeared on arms and legs, prompting re-initiation of itraconazole for 1 year before he was lost to follow-up. He denied any history of international travel.

Two years before admission, he developed prolonged fever, weight loss, hepatosplenomegaly, and pancytopenia along with persistent residual skin lesions. During this period, he was also diagnosed with pulmonary tuberculosis and received standard HRZE (isoniazid (H), rifampicin (R), pyrazinamide (Z), and ethambutol (E)) anti-tuberculosis treatment. Bone marrow biopsy revealed macrophages containing intracellular *Leishmania* amastigotes with kinetoplasts, confirming VL. He was treated with intravenous AmB-D for 1 month and continued oral itraconazole for another month before again being lost to follow-up. One year before admission, he developed progressive papulonodular skin lesions on both upper and lower limbs. Four months before admission, he experienced intermittent low-grade fever, anorexia, fatigue, and weight loss.

On admission, the patient’s vital signs were body temperature 38 °C, blood pressure 112/78 mmHg, heart rate 100 beats/min, and respiratory rate 22 breaths/min. Marked conjunctival pallor was observed. Active bleeding was present from the left nostril, with no visible mass detected on the nasal septum. Physical examination also revealed hepatosplenomegaly. Dermatological assessment revealed multiple infiltrative papulonodular lesions, including some wart-like growths of varying sizes, distributed across both upper and lower extremities, affecting dorsal hands, fingers, forearms, knees, and legs (Fig. 2F–I). Laboratory investigations showed pancytopenia (hemoglobin 4.6 g/dL, hematocrit 14.1%, white blood cell count 2,330 cells/mm^3^ (neutrophils 42%, lymphocytes 42%, eosinophils 6%, monocytes 10%), and platelet count 69,000 platelets/mm^3^).

**Fig. 3:**
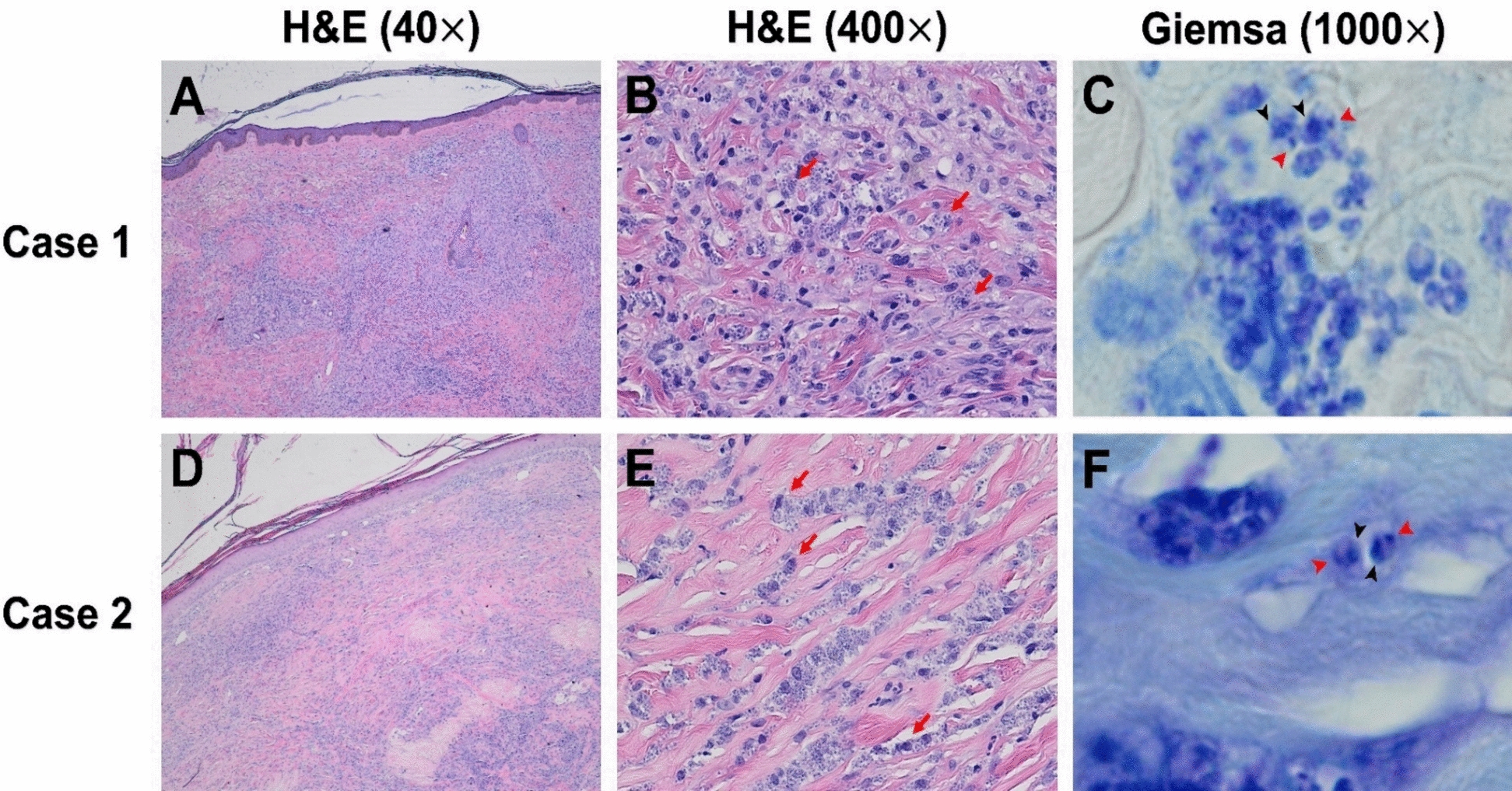
Histopathological features of Cases 1 and 2. **A**, **D** Hematoxylin and Eosin (H&E)-stained fibrotic dermal connective tissue with dense histiocytic infiltration. **B**, **E** Numerous intracellular amastigotes within macrophages (red arrows). **C**, **F** Giemsa stain confirms the presence of *Leishmania* amastigotes, each showing a nucleus (black arrowheads) and a kinetoplast (red arrowheads)

Bone marrow biopsy and aspiration, stained with Giemsa, revealed significant hypocellularity and numerous macrophages containing many amastigotes. Histopathological analysis of a forearm nodule showed fibrotic connective tissue with inflammatory cell infiltration and abundant intracellular amastigotes (Fig. 3A–C). *Leishmania martiniquensis* DNA was identified in the nodule biopsy, bone marrow, and whole blood, using a TaqMan-based duplex quantitative PCR (qPCR) assay targeting autochthonous species, as previously described [8]. Computed tomography of the neck was performed to investigate the cause of epistaxis and showed thickening of the nasal mucosa and septal membrane, consistent with mucocutaneous infection or inflammation. The patient was definitively diagnosed with concurrent diffuse CL and VL caused by *L. martiniquensis*, with suspected mucocutaneous involvement. After recovering from hemorrhagic shock, treatment commenced with intravenous AmB-D (1 mg/kg/day) for 1 month, followed by oral itraconazole (400 mg daily) for 1 month. The patient was advised to return for monthly AmB-D prophylaxis but declined and was lost to follow-up. Despite initial improvement, he was readmitted in February 2023 with high-grade fever, and he ultimately passed away.

### Case 2

**Fig. 4:**
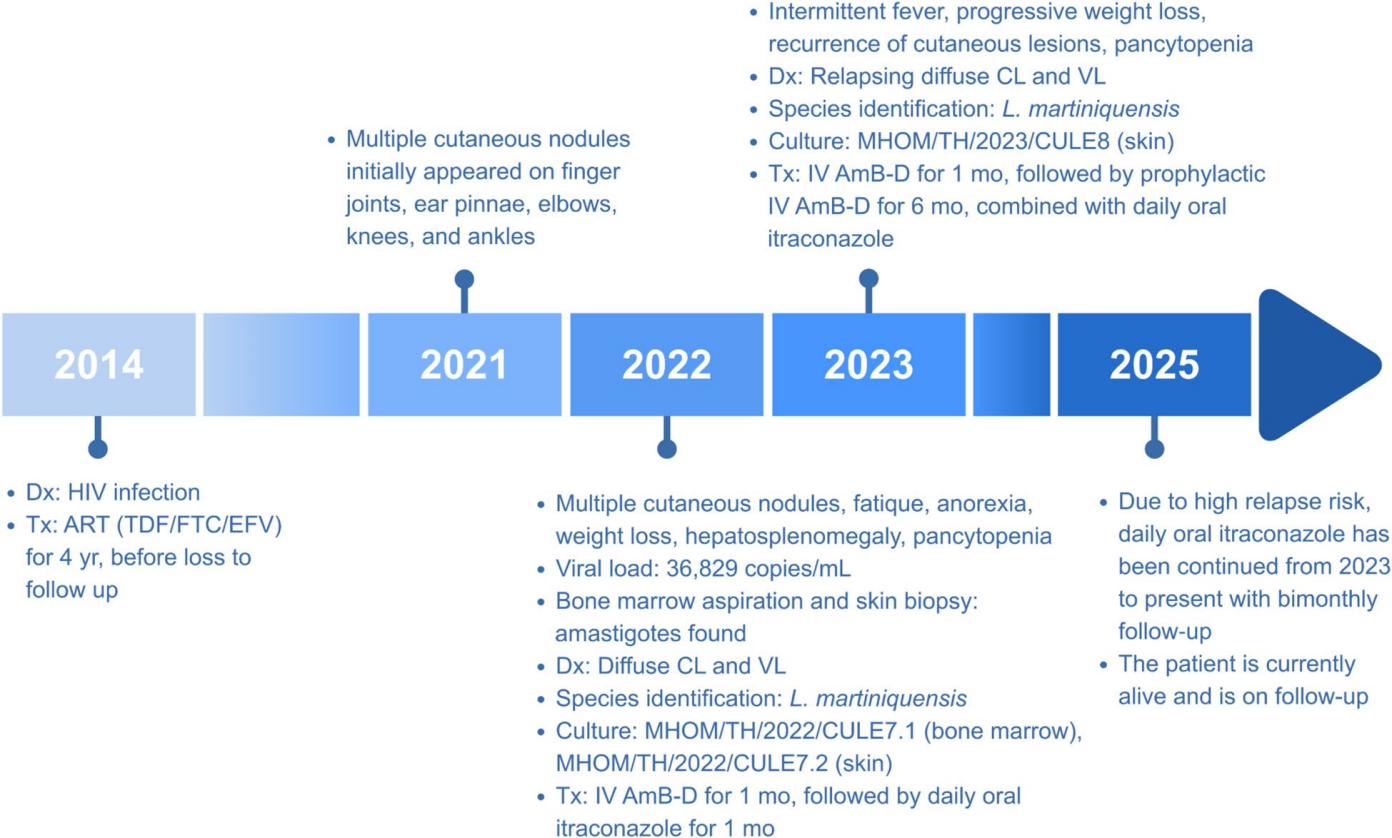
Key clinical timeline and management of Case 2 from 2014 to 2025

In December 2022, a 39-year-old male with AHD working as a general employee in Na Thawi District, Songkhla Province, Southern Thailand, presented with multiple cutaneous nodules, fatigue, malaise, anorexia, chronic diarrhea, and a significant weight loss of more than 5 kg over 1 month. One year before hospitalization, cutaneous nodules initially appeared on finger joints, ear pinnae, elbows, knees, and ankles, with no other systemic symptoms. Over the subsequent months, the nodules progressively enlarged and extended to palms and soles. He had been diagnosed with HIV in 2014 and received ART with tenofovir/emtricitabine/efavirenz (TDF/FTC/EFV) continuously since diagnosis, which he adhered to for 4 years before being lost to follow-up; adherence to HIV treatment thereafter was poor. Profound immunosuppression was evident from his recent HIV viral load of 36,829 copies/mL (September 2022), with CD4 count unavailable. There was no history of travel abroad. Figure 4 summarizes the key clinical events and management for this case.

**Fig. 5:**
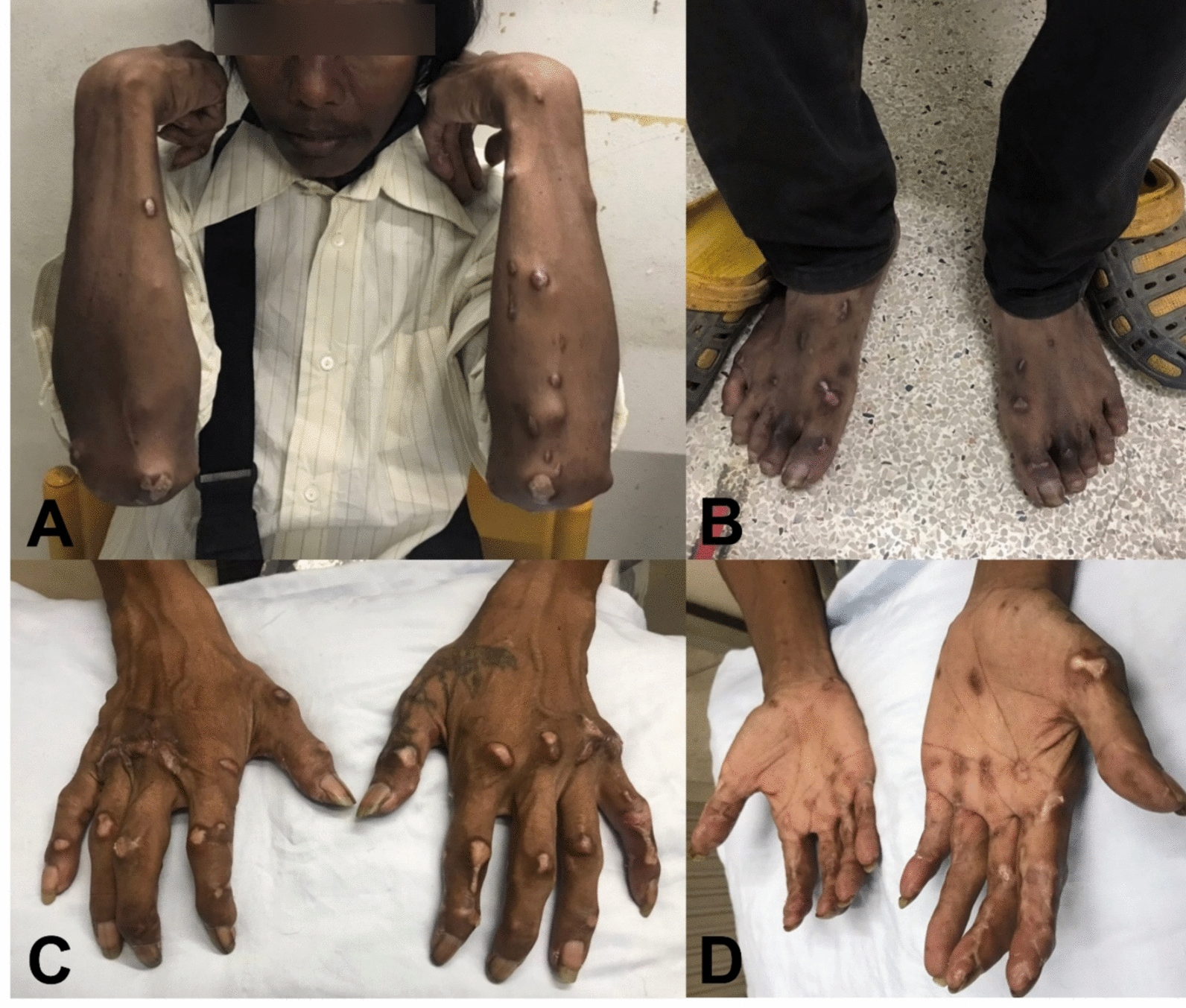
Cutaneous manifestations of Case 2. Multiple nodules on the extensor surfaces of both forearms (**A**), dorsal feet (**B**), dorsal hands/fingers (**C**), and palms (**D**)

**Fig. 6:**
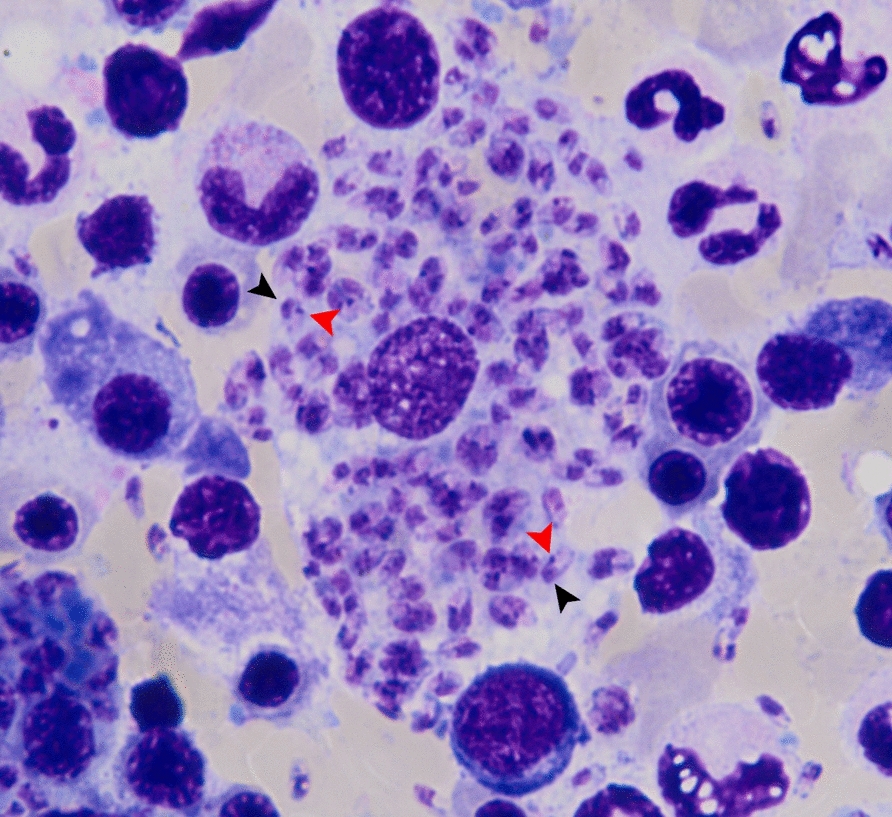
Representative Giemsa-stained bone marrow smear from Case 2 showing multiple *Leishmania* amastigotes within macrophages, each with a nucleus (black arrowheads) and a kinetoplast (red arrowheads)

On admission, vital signs were within normal limits, although mild conjunctival pallor and hepatosplenomegaly were observed. Physical examination revealed generalized multiple infiltrative nodules of various sizes affecting dorsal hands, especially at distal interphalangeal, proximal interphalangeal, and metacarpophalangeal joints, all fingers, as well as palms, forearms, arms, elbows, knees, legs, and dorsal aspects of the feet (Fig. 5A–D). Laboratory tests revealed pancytopenia (hemoglobin 9.2 g/dL, hematocrit 26.7%, white blood cell count 2,120 cells/mm^3^ (neutrophils 81%, lymphocytes 11%, monocytes 7%, basophils 1%), platelet count 114,000 platelets/mm^3^). Abdominal ultrasonography showed mild hepatomegaly with parenchymal changes and mild splenomegaly. Histopathological examination of a forearm nodule revealed fibrotic connective tissue with significant inflammatory cell infiltration and numerous intracellular amastigotes (Fig. 3D–F). Additionally, bone marrow aspiration was performed, and microscopic evaluation of a Giemsa-stained smear revealed macrophages parasitized by numerous intracellular amastigotes, consistent with VL (Fig. 6). *Leishmania martiniquensis* DNA was molecularly identified in both whole blood and saliva samples using the TaqMan-based duplex qPCR assay, as described elsewhere [8]. Promastigotes were successfully cultured from bone marrow and a skin biopsy. The qPCR analysis of these cultured promastigotes confirmed the identification of *L. martiniquensis*. They were assigned the WHO codes MHOM/TH/2022/CULE7.1 (bone marrow) and MHOM/TH/2022/CULE7.2 (skin). A definitive diagnosis of diffuse CL with subsequent VL involvement due to *L. martiniquensis* was established. Given his AHD-related immunosuppression and limited availability of alternative agents in our setting, the patient was treated intravenously with AmB-D at a dose of 1 mg/kg/day for 4 weeks, followed by oral itraconazole at 400 mg daily for 1 month. At the 1-month follow-up, significant improvement in cutaneous lesions was noted; however, the patient declined recommended monthly AmB-D prophylaxis.

However, 3 months later, he was readmitted with intermittent fever, progressive weight loss, and recurrence of cutaneous lesions that had previously improved but were now enlarging. Laboratory investigations again revealed pancytopenia. *Leishmania* promastigotes were cultured from a nodule biopsy and confirmed molecularly as *L. martiniquensis* (WHO code MHOM/TH/2023/CULE8). The full 4-week course of intravenous AmB-D was repeated, followed by prophylactic intravenous AmB-D (1 mg/kg/day for 5 days each month) for 6 months, in combination with daily oral itraconazole 400 mg, due to ongoing immunosuppression. For underlying HIV, ART with dolutegravir/lamivudine/tenofovir (DTG/3TC/TDF) was prescribed. After completing prophylactic AmB-D, significant clinical improvement was observed. Small nodules completely resolved, and large nodules significantly decreased in size. Due to high relapse risk, oral itraconazole 400 mg daily has been continued, with follow-up scheduled every 2 months. The patient is currently alive and is under regular follow-up.

## Discussion and conclusions

This report presents two cases of leishmaniasis caused by *L. martiniquensis* in patients with AHD, initially manifesting as diffuse cutaneous lesions that later progressed to visceral involvement. Diffuse CL represents a rare, anergic form characterized by numerous parasite-rich, non-ulcerative papules or plaques that initially appear on one body area before spreading widely, typically as nodules or plaques resembling lepromatous leprosy, within months [9, 10]. This presentation results from impaired host immunity due to underlying immunosuppression, such as advanced HIV, or direct parasite effects, resulting in transient treatment responses and frequent relapses [9]. The diffuse, infiltrative, non-ulcerative papulonodules observed in our immunosuppressed patients align with this classic diffuse CL definition, originally described before *L. martiniquensis* cases and typically associated with *Leishmania* species of the *Leishmania* subgenus, most commonly *L. aethiopica* and *L. amazonensis* [9, 10]. In our cases, *L. martiniquensis* progressed from diffuse cutaneous to visceral involvement, underscoring its broad clinical spectrum and poor prognosis in AHD. Beyond diffuse non-ulcerative lesions, *L. martiniquensis* can also cause a localized single papule or nodule (with or without ulceration) primarily on the head in immunocompetent individuals [11], or multiple ulcerative lesions on the trunk and extremities in immunocompromised patients [6, 12]. Recently, cases have been reported in patients with AHD where *L. martiniquensis* manifested concurrently as VL, diffuse CL, and MCL in the same individual, highlighting the parasite’s complex presentations [6].

Building on this clinical spectrum, the interaction between HIV and leishmaniasis creates a vicious cycle of mutual immunosuppression that drives such poor outcomes [13]. HIV-mediated CD4⁺ T-cell depletion impairs IFN-γ production by Th1 cells, thereby preventing macrophage activation essential for killing intracellular *Leishmania* amastigotes [14–16]. HIV further promotes Th2 cell differentiation, resulting in increased production of Th2 cytokines (IL-4, IL-10, TGF-β) that impair macrophage microbicidal activity, inhibit antigen presentation, and suppress Th1 differentiation, thus favoring parasite persistence and disease progression [14–16]. In turn, *Leishmania* infection further damages macrophage function and promotes HIV replication, yielding complex skin lesions, visceralization, treatment failure, high relapse rates, and elevated mortality, especially in VL-HIV co-infection [13–15]. Notably, ART is essential for restoring CD4 counts in patients with AHD and concurrent *Leishmania* infection [17]. However, in Case 1, persistently low CD4 counts despite ART likely reflect the synergistic immunosuppressive effects of *Leishmania* and HIV co-infection with high parasite burden.

This bidirectional interplay explains the cutaneous manifestations and disease progression observed in our cases, resulting from three synergistic factors: (1) profound AHD-related immunosuppression; (2) unique pathological features of *L. martiniquensis*; and (3) suboptimal treatment. First, advanced HIV infection with low CD4 counts (Case 1) or high viral load (Case 2) impaired Th1-mediated immunity, preventing effective parasite clearance and enabling the development of persistent non-ulcerative papulonodular lesions. Second, consistent with its known spectrum in HIV co-infection, *L. martiniquensis* manifested in our cases as chronic infiltrative papulonodular lesions with prominent fibrosis, as evidenced by our findings and a previous report [5], distinct from the classical ulceration typical of Old World cutaneous *Leishmania* species. Third, treatment interruptions from loss-to-follow-up promoted parasite persistence and relapse, exacerbating disease progression. Together, these factors not only underscore the diagnostic challenge of diffuse non-ulcerative lesions in patients with AHD but also emphasize the need for early diagnosis and optimized therapy in endemic regions.

The third factor, suboptimal treatment and resulting relapses, directly contributes to an emerging concern. The Thai case series documents high rates of AmB-D treatment failure after relapse [6]. Although Thai *L. martiniquensis* isolates from relapsed patients show significantly elevated IC_50_ values for AmB-D [18, 19], failure primarily results from synergistic immunosuppression and incomplete clearance, rather than resistance alone in advanced HIV co-infection. Under continuous drug pressure, incomplete parasite clearance can select for resistant subpopulations. Some *L. martiniquensis* isolates harbor genomic alterations linked to drug resistance, including a premature stop codon in the sterol C-24 reductase gene (disrupting ergosterol biosynthesis) and missense mutations in an ABC transporter-like gene (potentially mediating drug efflux), allowing resistant parasites to survive and dominate [19]. In both cases, patients with AHD failed to achieve parasite clearance after initial AmB-D therapy, resulting in relapse. This severe immunosuppression led to high parasite loads that overwhelmed host clearance, while ongoing AmB-D exposure exerted selective pressure for less susceptible parasites, promoting amphotericin B-resistant subpopulations, as evidenced by laboratory and clinical data [6, 19]. Parasite isolates collected from Case 2 before and after AmB-D treatment will be essential for future research into the mechanisms of AmB-D response and resistance.

These AmB-D resistance challenges directly underpin the difficulties in therapeutic management of *L. martiniquensis* infection, which remains complex due to limited treatment options and requires adherence to IDSA/ASTMH and WHO guidelines [17, 20]. For CL, localized therapies, such as heat therapy, cryotherapy, and intralesional antimonials, are preferred for uncomplicated lesions in immunocompetent patients. In contrast, systemic agents such as miltefosine, liposomal amphotericin B (L-AmB), AmB-D, pentavalent antimonials, pentamidine, or azole antifungals are essential for complex cases, including large, multiple, cosmetically or functionally critical, diffuse, disseminated, or immunosuppressed presentations as seen in our cases [20].

Mucocutaneous leishmaniasis, typically linked to New World *Leishmania* (*Viannia*) *braziliensis*, represents more severe mucosal involvement and requires prompt systemic therapy in all patients with metastatic disease to prevent disfigurement, airway obstruction, and death [20]. Recommended regimens align with CL systemic options, including pentavalent antimonials, AmB-D, L-AmB, or oral miltefosine, selected based on disease extent, tolerability, and comorbidities [20].

For VL, standard regimens in immunocompetent patients include L-AmB, AmB-D, miltefosine, and pentavalent antimonials [20]. For VL in patients living with HIV in South-East Asia, which is most relevant to our cases, the combination regimen is recommended to improve cure rates and limit resistance [17]. First-line therapy consists of L-AmB at a total dose of 30 mg/kg (5 mg/kg on days 1, 3, 5, 7, 9, 11) combined with miltefosine at 100 mg daily for 14 days [17]. If miltefosine is unavailable, monotherapy with L-AmB at a total dose of 40 mg/kg (5 mg/kg on days 1–4, 8, 10, 17, 24) is an acceptable alternative. Secondary prophylaxis is recommended for patients at high risk of relapse (e.g., no ART, CD4 < 200 cells/mm^3^, multiple VL episodes, or primary treatment failure), using AmB-D at 1 mg/kg administered as a single intravenous dose every 3–4 weeks or L-AmB at 3–5 mg/kg administered as a single intravenous dose every 3–4 weeks) [17].

While pentavalent antimonials are widely recommended as first-line therapy for Old World CL [21], these agents, including miltefosine, are currently unavailable in Thailand. In our resource-limited setting, where only AmB-D and off-label azoles are accessible due to the high costs of L-AmB, itraconazole was selected in combination with AmB-D based on previous Thai reports demonstrating efficacy against *L. martiniquensis*-HIV cases, achieving clinical regression despite profound immunosuppression [5, 6, 12]. This regimen deviates from guideline-based recommendations due to limited drug availability. Future strategies should prioritize expanded access to L-AmB, pentavalent antimonials, and miltefosine, along with close monitoring of ART adherence, to improve VL-HIV outcomes while mitigating the risk of resistance emergence.

Beyond therapeutic challenges, emerging evidence also extends traditional paradigms of leishmaniasis transmission in Thailand. Leishmaniasis has been traditionally known to be transmitted by phlebotomine sand flies. However, recent experimental evidence has demonstrated that members of the *Mundinia* subgenus, including *L. martiniquensis*, can successfully develop to the infectious metacyclic promastigote stage in the midgut of the biting midge *Culicoides sonorensis* [22]. Moreover, *Leishmania* has been molecularly detected at high prevalence in *Culicoides* midges collected near patients’ residences in endemic regions of Thailand [23–25]. Together, these findings support *Culicoides* midges as the likely vectors of autochthonous leishmaniasis and underscore the need for further entomological investigations to confirm vector competence and clarify the unique transmission dynamics of *Mundinia* parasites in Thailand.

In summary, these cases highlight the cutaneous presentations and therapeutic challenges of *L. martiniquensis* in Thai patients with AHD. Beyond classical ulcerative lesions seen in other *Leishmania* species, *L. martiniquensis* can manifest as diffuse, non-ulcerative, infiltrative papulonodular lesions, particularly in immunocompromised hosts, with a propensity for progression to visceral or mucocutaneous disease [6]. Clinicians should maintain a high level of suspicion for these complex cutaneous forms, pursue early parasitological confirmation, and initiate timely and effective therapy to improve patient outcomes and mitigate the spread of drug-resistant parasites in Thailand.

## Data Availability

No datasets were generated or analyzed during the current study.
